# Parallel targeted and non-targeted quantitative analysis of steroids in human serum and peritoneal fluid by liquid chromatography high-resolution mass spectrometry

**DOI:** 10.1007/s00216-022-03881-3

**Published:** 2022-01-19

**Authors:** Thomas Andrieu, Therina du Toit, Bruno Vogt, Michael D. Mueller, Michael Groessl

**Affiliations:** 1 Department of Biomedical Research (DBMR), University of Bern, Bern, Switzerland; 2 Department of Gynecology and Gynecological Oncology, Inselspital, Bern University Hospital, University of Bern, Bern, Switzerland; 3 Department of Nephrology and Hypertension, Inselspital, Bern University Hospital, University of Bern, Freiburgstrasse, 3010 Bern, Switzerland

**Keywords:** LC–MS, High resolution, Steroids, Serum, Peritoneal fluid, Menstrual cycle, Dienogest

## Abstract

We developed and validated a liquid chromatography high-resolution mass spectrometry method for the absolute quantification of 51 steroids for clinical analysis of human serum and, for the first time, peritoneal fluid. Data acquisition was performed in both targeted and untargeted mode simultaneously, thus allowing the accurate and precise quantification of the main components of the classical steroid pathways (17 steroids) as well as the analysis of 34 additional non-classical steroids. For targeted analysis, validation was performed according to FDA guidelines, resulting, among other parameters, in accuracy < 13% RSD and precision < 10% relative error, for both inter- and intra-day validation runs. By establishing steroid-specific response factors, the calibration curves of the targeted analytes can be extended to untargeted analytes. This approach opens novel possibilities for the post hoc analysis of clinical samples as the data can be examined for virtually any steroid even after data acquisition, enabling facile absolute quantification once a standard becomes available. We demonstrate the applicability of the approach to evaluate the differences in steroid content between peripheral serum and peritoneal fluid across the menstrual cycle phases, as well as the effect of the synthetic gestagen dienogest on the steroid metabolome.

**Graphical abstract:**

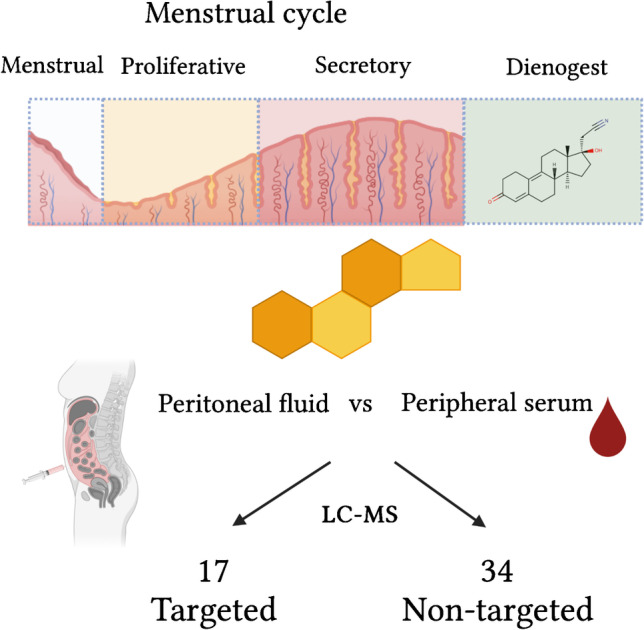

**Supplementary Information:**

The online version contains supplementary material available at 10.1007/s00216-022-03881-3.

## Introduction

Steroids represent a subgroup of the metabolome; they are small and highly important signaling molecules and their metabolism is complex [1]. All steroids exhibit the same core structure which consists of three six-membered and one five-membered rings. The steroid core structure can be modified in almost all positions in either oxidation state (formation of double bonds) or addition of functional groups, e.g., hydroxyls, leading to a large number of structurally similar compounds with diverse biological functions. Steroids are involved in a plethora of biochemical processes, and their identification and quantification is therefore crucial for understanding steroid-mediated signaling which is of vital importance for fundamental physiological processes such as sexual differentiation; reproduction; fertility; hypertension; homeostasis; and initiation, promotion, and progression of certain types of cancer [1–3]. Consequently, steroidogenic enzymes and steroid receptors present very attractive drug targets and numerous steroid biosynthesis inhibitors are in clinical use. Additionally, steroids represent excellent biomarkers for disease [4].

Liquid chromatography-mass spectrometry (LC–MS)–based methods have become the gold standard for the clinical analysis of steroids in human serum and urine [5–9]. For example, Eisenhofer et al. studied the impact of gender, age, oral contraceptives, body mass index, and blood pressure status on the level of 16 adrenal steroids in plasma in over 500 subjects [5]. Analysis of urinary steroid metabolites by ultra-high pressure liquid chromatography coupled to high-resolution accurate mass-MS (UHPLC-HRAM-MS) has also recently been reported by Singh et al. for the reliable diagnosis of adrenal disorders such as adrenocortical adenoma and carcinoma [8]. Current clinical MS-based serum steroid panels consist of fewer than 15 steroids, usually due to technical difficulties or the lack of knowledge on the biological and clinical relevance of additional steroids. Also, steroids are usually measured in targeted mode, i.e., no steroids other than the ones pre-defined before measurement can be quantified.

In addition to serum, other biological fluids such as peritoneal fluid (PF) also contain steroids. Quantifying steroids in such fluids might have the advantage of giving a more “local” representation of steroid metabolism, in contrast to the systemic information provided by serum in which dysregulations in specific organs might be “diluted” and therefore difficult to detect.

The peritoneal cavity is a compartment within the abdomen that is limited by the peritoneum, a semi-permeable membrane lining the abdominal and visceral organs and includes female reproductive organs (ovaries and uterus). A physical connection between the uterus lumen and the peritoneal cavity is established through the fallopian tubes. The uterus structure is strongly affected by the ovarian hormones estradiol (E2) and progesterone (P4), produced during the proliferative phase and the secretory phase, respectively. In absence of fecundation, the corpus luteum regresses leading to P4 withdrawal, endometrium involution, and finally menstruation bleeding. The peritoneal cavity works like a receptacle, and PF is produced regularly [10]. Indeed, the peritoneum is important in osmoregulation; it helps to maintain osmotic and chemical equilibrium with blood and lymph. PF is derived by (a) transudation from blood plasma; (b) exudation from ovarian surface tissues; (c) transudation or exudation from intra-abdominal organs not classified as part of the reproductive system, such as the kidneys, liver, pancreas, and intestines; and (d) contributions from intra-abdominal fat. Its analysis is used for the diagnosis of cirrhosis, peritonitis, pancreatitis, and malignancy. In addition, retrograde menstruation, a common process in which the menstrual blood accesses the peritoneal cavity through the fallopian tube is another source for PF. PF can be collected during laparoscopy at the rectovaginal area using a simple syringe.

Reports on steroids present in PF are rather sparse [10–13]. Yet, steroids might serve as good predictors in diseases such as endometriosis or ovarian cancer: LC–MS applied to ectopic lesions revealed a specific pattern for P4 and testosterone (T) compared to serum [14]. In endometrial cancer, blood steroid levels predict survival in endometrial cancer and reflect tumor estrogen signaling [15]. In ovarian cancer, steroid hormone synthesis by the ovarian stroma surrounding epithelial ovarian tumors may participate in ovarian tumorigenesis [16]. Circulating 17α-hydroxyprogesterone (17OHP4) and 11-deoxycortisol (S) have been suggested as prognostic markers for endometrial cancer survival [15]. Finally, T was positively associated with epithelial ovarian cancer risk [17].

**Fig. 1:**
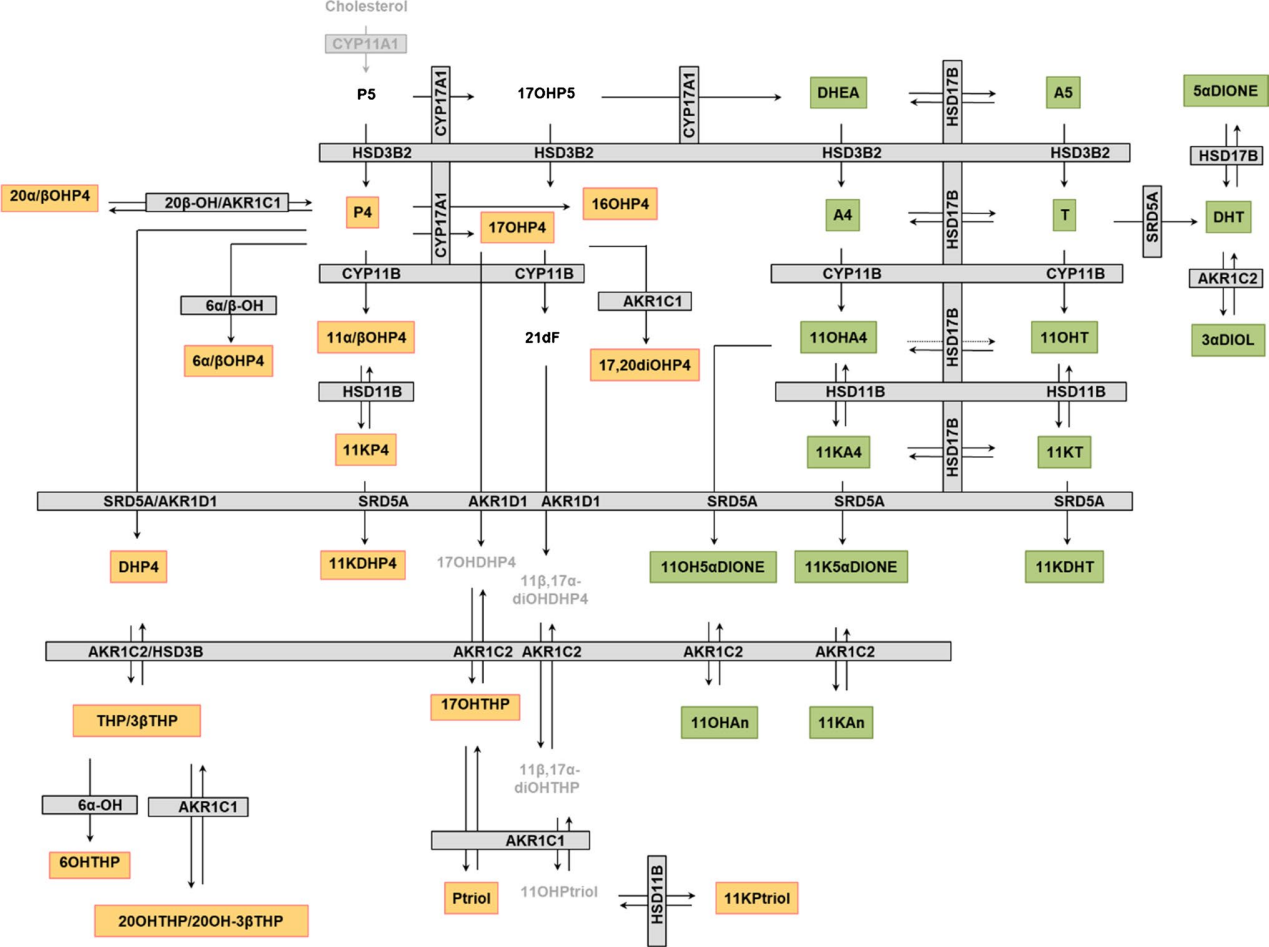
Metabolic pathways of progesterones (orange boxes) and androgens (green boxes) investigated in this study. The remaining steroids belong either to other classes (black) or were not measured (grey). Enzymes are displayed in grey boxes. See Supporting Information for a list of all 51 investigated steroids including the used abbreviations

We therefore set out to establish an LC–MS method that not only allows both the targeted analysis of “classical” steroid pathways but also allows post hoc untargeted absolute quantitation of virtually any other steroid that might be of interest and for which a standard is available. We applied this method in both serum and PF to cover the majority of metabolic pathways of potential interest including progesterones and C11-oxyandrogens (Fig. 1).

## Materials and methods

### Chemicals and reagents

Formic acid, water, methanol, and acetonitrile (all LC–MS grade) were from Biosolve (Switzerland). Zinc sulfate heptahydrate was obtained from Sigma-Aldrich (Switzerland); double charcoal stripped, delipidized human serum was obtained from Golden West Diagnostics (USA). Steroid standards for the targeted workflow were obtained as certified reference solutions from Cerillant (UK), all other from Steraloids (USA).

### Clinical samples

The study has been approved by the Swiss Ethics Committee (KEK-BE 149/03, 45 2003), and all participants provided written informed consent. Eighty-two women in reproductive age from 18 to 45 years were recruited for this study. PF was collected during laparoscopy; serum was obtained prior to anesthesia. To determine the steroid signature specific for each cycle phase, women who were uncycled, who presented an unclear cycle, or who had irregular bleeding (metrorrhagia and menometrorrhagia) were excluded from the analysis. Women using contraception, intrauterine devices, or under hormonal treatment other than Visanne (dienogest) were not retained. All women under Visanne started the treatment at least 3 months before the sample collection. PF samples with a total protein content < 15 mg/mL, as determined using a micro-bicinchoninic assay (Quanti-Pro BCA, Sigma-Aldrich, USA), were excluded to ascertain absence of dilution with abdominal flushing medium under the clinical procedure. Hemolytic samples were excluded from the study. Finally, patients with prior or current infections and liver dysfunction were excluded from the study.

Forty-seven patients were therefore retained (Table 1). Among them, 16 were in proliferative phase, 11 in secretory phase, and 11 in the menstruation phase and 9 using hormonal medication (dienogest).

**Table 1:** Characteristics of the patient cohort

	Menstruation	Proliferative	Secretory	Dienogest
*Number of patients*	11	16	11	9
*Age (year, SEM)*	35.0 ± 1.4	36.8 ± 1.5	35.1 ± 1.6	31.9 ± 2.7
*Time blood draw (h, SEM)*	10.2 ± 0.6	12 ± 0.7	10.1 ± 0.9	11.2 ± 0.7
*Peritoneal fluid (mL, SEM)*	6.4 ± 1.7	7.0 ± 1.4	14.6 ± 2.6	9.1 ± 2.0
*Cycle length (day, SEM)*	29.6 ± 2.0	27.2 ± 0.5	26.1 ± 0.7	n/a
*Period length (day, SEM)*	5.3 ± 1.0	5.6 ± 1	4.8 ± 0.3	n/a

### Liquid chromatography high-resolution mass spectrometry

All calibrants and QC samples were prepared in double charcoal stripped, delipidized human serum: see Supporting Information for a list of concentrations. Five hundred microliters of the sample (serum or PF) was spiked with 38 μL of a mixture of isotopically labeled standards in methanol (3.8 nM each). Two hundred fifty microliters of zinc sulfate (0.1 mol/L) and 500 μL of cold methanol (− 20 °C) were added; samples were vortexed and centrifuged for 5 min at 8000* g*. Two hundred fifty microliters of water was added to each sample and purified using solid-phase extraction (SPE) on an OasisPrime HLB 96-well plate using a positive pressure 96-well processor (both Waters, UK). Samples were eluted using pure acetonitrile which was subsequently dried under nitrogen. Samples were resuspended in 100 μL of 33% methanol in water. Twenty microliters were injected into the liquid chromatography high-resolution mass spectrometry (LC-HRMS) instrument (Vanquish UHPLC coupled to a QExactive Orbitrap Plus; both Thermo Fisher Scientific, Switzerland). Separation was achieved using an Acquity UPLC HSS T3 column, 100 Å, 1.8 μm, 1 mm × 100 mm (Waters, UK). Mobile phases A and B consisted of water + 0.1% formic acid and methanol + 0.1% formic acid, respectively. The separation method was as follows (constant flow of 0.15 mL/min): 0–0.5 min 1% B, 0.5–1 min linear gradient to 1–46% B, 1–4 min 46%, 4–12 min linear gradient 46–73% B, 12–12.5-min linear gradient 73–99% B, 12.5–14.5 min 99% B, 14.5–15-min linear gradient to 1% B, 15–17 min 1% B. The mass spectrometer was operated both in negative and positive ion modes using an electrospray ionization source (spray voltage of 4.5 kV in positive and 4 kV in negative ion modes), an inlet capillary temperature of 250 °C, and an aux gas heater temp of 300 °C. Sheath gas flow rate was set to 40, aux gas flow rate to 10, and sweep gas flow rate to 2. S-lens RF-level was set to 80. The instrument was operated in both parallel reaction monitoring (PRM) and full scan mode acquiring full scan data MS resolution setting of 70′00 for full scan and 17′500 for PRM) in a mass range of 200–500 m/*z* (AGC target 1e6 and maximum injection time 100 ms). See Table S1 for a list of monitored masses and transitions. For data analysis, tolerance of all *m*/*z* values was set to 5 mmu. All data were processed using TraceFinder 4.0 (Thermo Fisher Scientific, Switzerland).

### Method validation of targeted analytes

The targeted method was validated according to FDA guidelines for bioanalytical method validation [18] for the following parameters: lower and upper limit of quantification (LLOQ und ULOQ), range, accuracy, precision, carryover, recovery, robustness, and stability (Supporting Information Tables S3–S8).

The LLOQ and range of the assay were calculated using replicates (*n* = 6 at each level) of double charcoal stripped, delipidized human serum at four different concentrations (QC samples LLOQ, low, mid, and high) on three different days. As different values are expected for each compound in clinical samples, the QC values were selected specifically for each analyte (see Supporting Information Table S2). A 12-point calibration was performed for all analytes covering the range from the lowest to the highest QC concentrations. A blank (no matrix, no internal standards), a matrix blank (matrix, no internal standards), and a zero calibrator (matrix and internal standards) were included in all calibrations. Intra-assay accuracy and intra-assay precision were calculated based on the replicate determination of each concentration level made on three separate days (*n* = 6 at each concentration and on each day). The carryover was determined by analyzing a solvent sample (33% methanol) after injection of the calibrant at the highest concentration on each day. Robustness was assessed by performing an additional set of accuracy and precision experiments by a different technician using different lots of solvents, SPE plate, and LC column (*n* = 6 at three concentration levels low, mid, and high).

Different stability parameters were evaluated: freeze–thaw stability of unprocessed biological samples (3 freeze–thaw cycles, min. of 12 h between each cycle), bench-top stability of unprocessed biological samples (24 h), long-term storage of unprocessed biological samples at – 20 °C (3 months), and autosampler stability (4 °C) of processed biological samples (48 h). QC samples (*n* = 6 at 3 the different QC levels low, mid, and high) and serum samples of six healthy volunteers were used for this purpose. Dilution linearity was tested by 1:4 dilution of these samples in phosphate-buffered saline solution prior to processing.

### Untargeted analysis

Individual calibration curves of all other steroids of interest (see Supporting Information Table S9) covering the concentration range from 0.1 to 2000 nM were also prepared in double charcoal stripped, delipidized human serum and processed as described above. These steroids were only measured in full MS mode. Calibration curves of untargeted analytes were compared to androstenedione (A4) and P4 which allowed to establish correction factors due to different ionization efficiencies by linear regression.

In addition to steroid concentrations, we also calculated apparent activities of steroid-metabolizing enzymes by calculating the corresponding product-to-substrate ratio. The steroid pathways including both the steroids and the enzymes are depicted in Fig. 1, and all analytes and ratios are listed in the Supporting Information (Table S10). Statistical analyses were performed using MetaboAnalyst 5.0 [19] and Prism 8 (GraphPad Software, USA). Prior to pathway analysis, data was logarithmized and normalized to standard score (*z* score).

## Results

### Method validation

Our analytical workflow consists of three steps: protein precipitation using zinc sulfate and methanol, steroid purification using SPE, and analysis using LC-HRMS. The sample to sample time is 17 min, and 17 steroids are absolutely quantitated by the aid of analyte-specific isotopically labeled internal standards.

We carried out method validation according to the FDA guidelines on bioanalytical method validation (see “Materials and methods” for details). The range of our method defined by the lower and upper limits of quantification (LLOQ and ULOQ) is analyte-dependent as the physiological concentrations of different steroids vary strongly. In general, LLOQs lie in the range of approximately 100 pmol/L and ULOQs in the range of 1000 nmol/L; for details, please refer to Table 2. For all concentrations at the LLOQ, accuracy and precision were found to be below 20% relative standard deviation (RSD) and relative error, respectively, as required by the FDA validation guidelines [18]. Also, at the other concentration levels, the requirements of the guideline (accuracy and precision < 15% RSD and relative error, respectively) were fulfilled for both inter- and intra-day validation runs (Tables 2 and S3). During carryover assessment as tested by running a solvent sample after the highest calibration standard, detected peaks (if any) were lower than 20% of the signal at LLOQ. Robustness was assessed by performing an additional set of accuracy and precision experiments by a different technician using different lots of solvents, SPE plate, and LC column at three different concentrations; accuracy was found to vary between 1.3 and 12.2% RSD, whereas accuracy was between − 8.9 and 14.2% relative error (Table S4).

**Table 2:** Assay range, accuracy, and precision of inter-day validation runs for targeted analytes

	Range (nmol/L)		Accuracy (RSD, %)				Precision (relative error, %)			
	LLOQ	ULOQ	High	Mid	Low	LLOQ	High	Mid	Low	LLOQ
11-Deoxycorticosterone	0.092	189	5.7	4.7	5.7	10.1	− 1.2	− 0.2	0.9	0.3
11-Deoxycortisol	0.088	180	5.2	6.4	5.8	7.2	− 1.3	− 4.8	− 1.5	− 1.9
17α-Hydroxyprogesterone	0.092	189	6.9	8.9	5.7	9.6	− 1.9	− 1.2	0.2	− 5.7
21-Deoxycortisol	0.088	180	7.9	7.3	6.3	16.3	− 3.5	− 2.6	2.2	− 6.2
5α-Dihydrotestosterone	0.105	215	6.4	5.9	8.1	9.6	− 0.6	− 1.9	5.7	− 8.4
Aldosterone	0.085	173	14.9	6.7	9.7	10.3	9.5	3.9	3.3	3.1
Androstenedione	0.107	218	5.4	5.2	6.9	7.7	− 4.8	0.1	− 2.0	6.9
Androsterone	0.420	861	9.3	9.4	8.2	10.5	− 7.0	− 3.7	2.7	8.4
Corticosterone	0.705	1443	5.1	7.0	5.3	7.6	0.2	-3.3	2.3	9.8
Cortisol	0.378	8967	8.3	10.3	7.6	14.6	2.2	0.9	− 1.9	5.6
Cortisone	0.177	1387	5.9	5.6	5.2	4.6	− 0.2	− 2.9	0.9	5.5
DHEA	0.846	867	10.8	8.0	12.8	16.6	0.1	− 1.4	2.7	9.6
DHEA-S	6.252	12,805	8.8	7.4	7.5	7.0	2.1	7.4	4.3	4.6
Etiocholanolone	0.210	215	6.5	6.0	6.1	16.6	− 3.7	0.0	1.9	0.6
Pregnenolone	0.771	790	5.5	6.1	9.3	8.5	− 0.2	− 5.9	7.7	− 5.9
Progesterone	0.476	1590	6.2	5.2	4.5	6.3	0.3	− 2.2	1.7	2.9
Testosterone	0.105	867	6.2	6.2	5.8	7.4	− 3.2	− 3.8	− 1.1	3.9

Additionally, we evaluated four different stability parameters at three different concentration levels: freeze–thaw stability (3 cycles), bench-top stability of unprocessed biological samples (room temperature; 24 h), long-term storage at – 20 °C (3 months), and autosampler stability (48 h). All analytes were stable under these conditions as evidenced by accuracy (maximum 14.6% RSD) and precision (maximum 14.7% relative error). Analyte recovery was > 80% in all cases (see Tables S5–S8 (Supporting Information) for details).

### Untargeted analysis

After having performed the targeted analysis for 17 steroids as described above, we applied our non-targeted workflow to the LC–MS raw data. For this purpose, we established a database of 34 additional steroids containing experimental mass spectra and retention times obtained from authentic standards and used these parameters to examine the LC–MS data for these compounds. Having established the response factors of all 34 steroids relative to steroids in the targeted workflow, we were able to also perform absolute quantitation for these analytes (see Supporting Information for examples of extracted ion chromatograms, Fig. S3). For example, to determine the concentration of 11-ketoandrostenedione (11KA4), we use the calibration data for A4 from the targeted workflow and correct it with the specific correction factor for this compound (multiplication of the concentration with 0.329 for 11KA4). The correction factors for all investigated compounds are listed in Table S9 (Supporting Information). From a physicochemical point of view, the correction factor reflects the difference in ionizability between the two compounds during the electrospray process. This approach is valid as the calibration curves are linear over four orders of magnitude and the correction factor therefore remains constant over a large concentration range as shown by linear regression analysis (Supporting Information). There are two major advantages of such a surrogate calibration: only a small number of calibrants needs to be measured for the quantification of a large number of analytes and the panel of analytes can be assembled post hoc depending on the biological question at hand. Still, it has to be noted that the accuracy suffers from this approach as no specific isotopically labeled internal standard is available for each analyte. Therefore, matrix effects cannot be corrected as efficiently as for the targeted analytes.

In order to assess how much error is introduced, we performed correlation analysis on the results from targeted and untargeted analysis for 9 different analytes in 22 clinical samples. The results for these 9 analytes are shown in the Supporting Information (Fig. S1).

As 13 different internal standards are included in each sample covering the entire range of elution times, the error remains relatively small. The offset is usually negligible with < 10% of the median analyte concentration. However, based on the slope of the regression curve, a constant bias in the range of 10–30% may be introduced. Note that this approach of surrogate calibration can be extended to any analyte for which a constant correction factor to one of the targeted analytes can be established using an authentic standard.

### Steroid profiles: PF versus serum

**Fig. 2:**
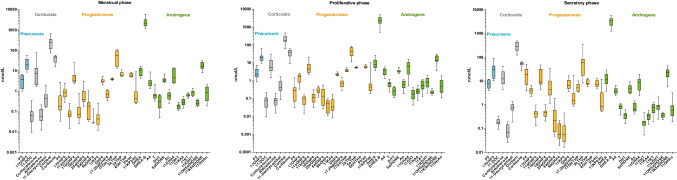
Concentrations of steroids in serum in dependence of the menstrual cycle

**Fig. 3:**
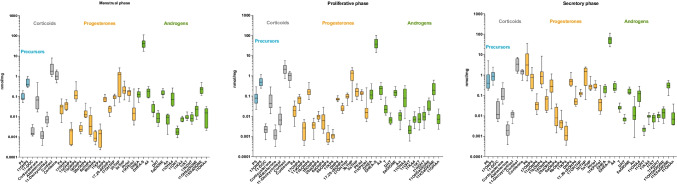
Concentrations of steroids in PF in dependence of the menstrual cycle

Figures 2 and 3 show the quantified steroid levels in serum and peritoneal fluid according to the menstrual phase. Additionally, the levels of each steroid as well as the apparent enzyme activities for each group across the menstrual phase and under dienogest treatment are reported and compared in the Supporting Information (Table S10). Most notably, we quantify for the first time in PF the C11-oxy C_19_ androgens in the following order: 11β-hydroxy-5α-androstane-3,17-dione (11OH5αDIONE) > 11-ketotestosterone (11KT) > 11KA4 > 11β-hydroxyandrostenedione (11OHA4) >≈11β-hydroxytestosterone (11OHT), while 11OHA4, 11KT, and 11OHT were present in serum at higher concentrations than in PF.

We found that majority of the levels measured in PF correlated strongly (*p*-value of regression analysis < 0.05 for 37 out of 51 steroids) with the concentration measured in the serum. In general, mineralocorticoids, androgens, and glucocorticoids are more highly concentrated in serum (about twofold on average), whereas progesterone metabolites are higher in PF (2–threefold on average). Levels of A4 and its 5α-reduced product, 5α-androstane-3,17-dione (5αDIONE), together with 11OH5αDIONE, were present at significantly higher levels in PF than in serum, indicating 5α-reductase (SRD5A) activity in the organs connected to the peritoneal cavity such as the ovaries.

The serum-to-PF ratio of mineralocorticoids and glucocorticoids is not affected by the menstrual cycle, in contrast, to androgens and progestogens. During secretory phase, the PF is dominated by progesterones as all downstream progesterone metabolites were quantified at high levels. In addition, the serum/PF ratio increases for androgens while decreasing for progesterones. Essentially, this shows that the concentration of steroids is primarily governed by their origin: steroids produced by the ovaries such as the progestogens P4, 17OHP4, A4, and pregnenolone (P5) are higher in PF, while the adrenal steroids are higher in serum.

Looking at apparent enzyme activities calculated as product-substrate ratios, increased 11βHSD2 activity is observed in PF compared to serum (cortisone/cortisol, 11KA4/11OHA4, and 11KT/11OHT ratios). Whereas one-third of the apparent enzyme activities related to the progestagen pathways were affected by the cycle phase, only one corticosteroid-related enzyme (cytochrome P450 21-hydroxylase, CYP21A2) and two androgen-related enzymes showed strong cycle dependency (17β-hydroxysteroid dehydrogenase, HSD17B and SRD5A).

**Fig. 4:**
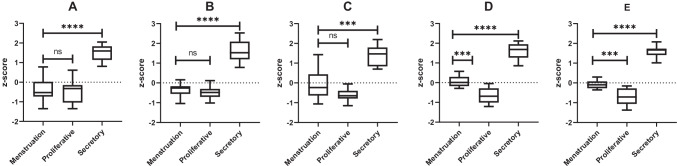
Comparison of different classifiers for the determination of the menstrual cycle phase. **A** Progesterone levels in serum. **B** Progesterone levels in PF. **C** Combined serum classifier: (20αOHP4)/(CYP17A1 + HSD3B2); (20αOHP4: 20α-hydroxyprogesterone). **D** Combined PF classifier: (5αDHP4 + 20αOHP4)/CYP17A1. **E** Combined serum + PF classifier: (serum 5αDHP4 + serum 20αOHP4 + PF 20αOHP4)/PF CYP17A1; (5αDHP4: 5α-dihydroprogesterone). CYP17A1: 17OHP4/P4. HSD3B2: P4/P5. Ns, not significant. ***p-value < 0.001. ****p-value < 0.0001

Usually, P4 and E2 levels are used for determination of the proliferative and secretory phases of the menstrual cycle. Due to technical limitations (low ionizability combined with picomolar concentration), E2 is not measurable in multi-steroid assays as derivatization is required to improve detection limits; yet, derivatization leads to more complex sample preparation, might interfere with the detection of other steroids and requires specialized LC–MS conditions, and is therefore not compatible with the parallel analysis of the other steroid classes [20]. We therefore investigated if other steroids in our assay might serve as markers for the menstrual cycle phases. In addition to single steroids, we also evaluated apparent enzyme activities and combinations thereof. We then compared the best performing classifiers in PF and serum to P4 alone (Fig. 4).

Whereas P4 clearly differentiates the secretory phase in both serum and PF, no significant differences between menstruation and proliferative phase exist. On the contrary, if a multi-component classifier is constructed by the combination of multiple steroids and apparent enzyme activities, also these two phases can be differentiated. Whereas the performance of the combined serum classifier is not very strong, the combined PF classifier shows better performance. Best performance is obtained if data from both PF and serum is combined.

### Steroid pathways

**Fig. 5:**
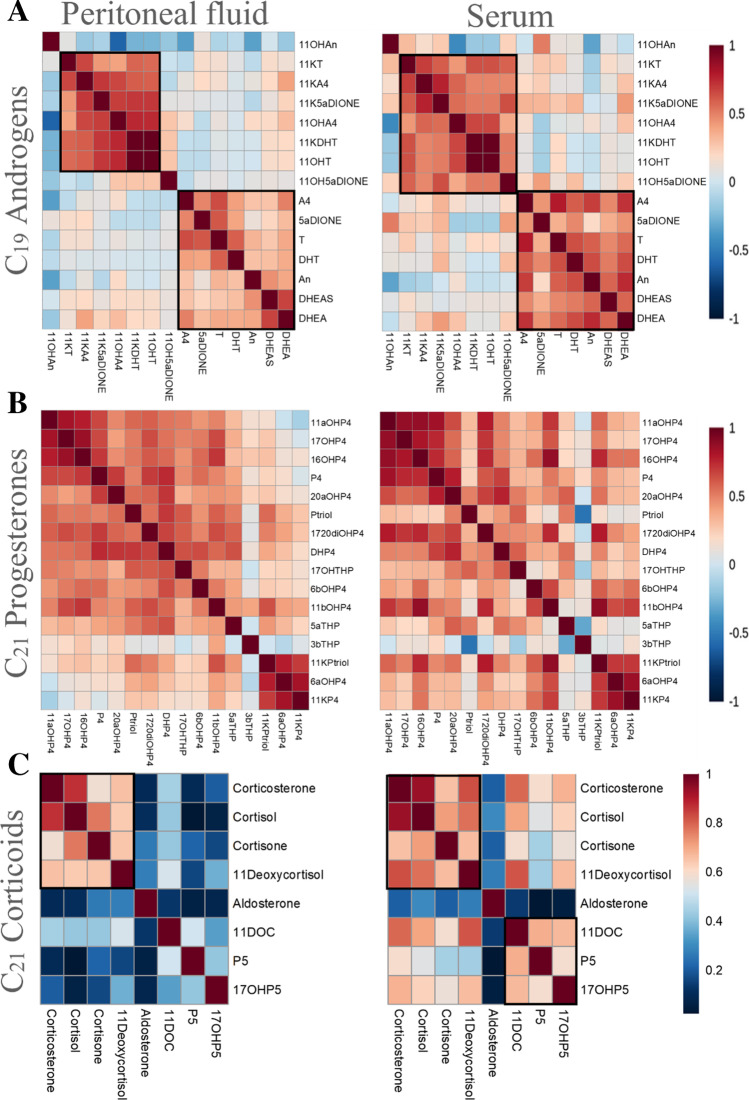
Correlation heat maps showing Spearman’s rank correlation coefficient of **A** C_19_ androgens, **B** C_21_ progesterones, and **C** C_21_ corticoids in PF (left panels) and in serum (right panels). Black boxes visually identify steroids clustered based on strong correlations

The heat maps presented in Fig. 5 illustrate Spearman’s rank correlation coefficients between the steroid concentrations in PF and serum according to steroid class. These heat maps allow facile identification of active androgen, progesterone, and corticoid pathways.

**Fig. 6:**
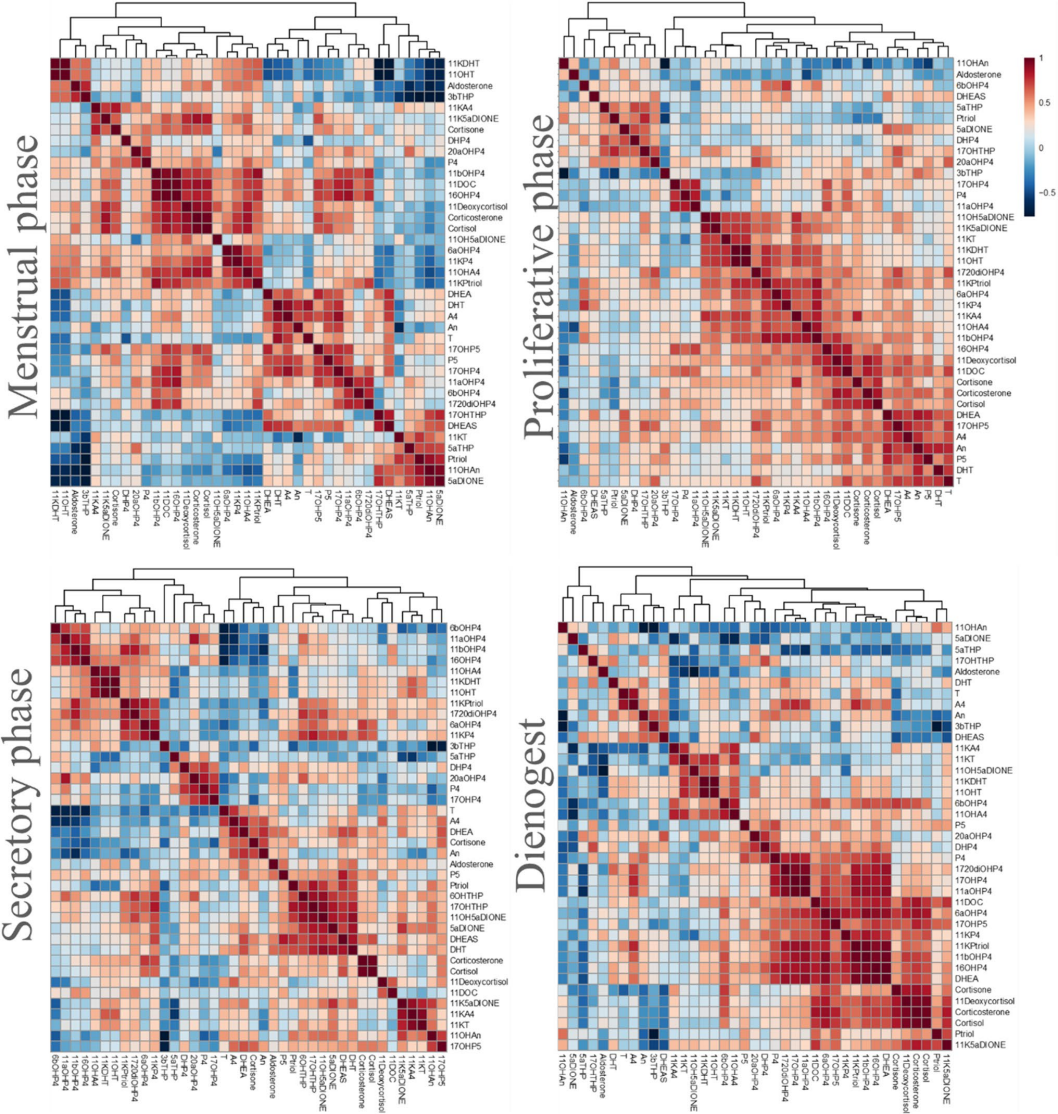
Correlation heat maps showing the correlation of all the quantified steroids (Spearman correlation) in serum during the menstrual phase, proliferative phase, secretory phase, and under dienogest treatment

In PF (Fig. 5A), a correlation cluster comprising the C11-oxy androgens 11-ketoandrostanolone (11KDHT), 11KT, 11KA4, 5α-androstanetrione (11K5αDIONE), 11OHT, and 11OHA4 was identified. Additionally, testosterone (T) correlates strongly with dihydrotestosterone (DHT) and 5αDIONE (Fig. 6A). In serum, the same correlation cluster as in PF was identified for C11-oxy androgens. The “classical” androgens A4, DHT, T, androsterone (An), dehydroepiandrosterone (DHEA), and DHEA-S correlate much more strongly in serum compared to PF.

For progesterones (Fig. 5B), a large correlation cluster consists of P4 and its direct metabolites (produced through only one enzymatic step), including 11α-hydroxyprogesterone (11αOHP4); 11β-hydroxyprogesterone (11βOHP4); 17OHP4; 16α-hydroxyprogesterone (16αOHP4); 5α/βDHP4; and 17α,20α-dihydroxyprogesterone (17,20diOHP4) in both serum and PF (Fig. 5B). Note that the progesterone correlation maps are strongly influenced by the presence of high progesterone production during the secretory phase. In both PF and serum, an additional correlation cluster of the far downstream metabolites 11-ketopregnanetriol (11KPtriol), 6α-hydroxyprogesterone (6αOHP4), and 11-ketoprogesterone (11KP4) was identified.

For the corticoids in PF (Fig. 5C), only corticosterone, cortisol, cortisone, and 11-deoxycortisol are strongly correlated, whereas strong correlations between all corticoids with the exception of aldosterone are observed in serum (Fig. 5C).

Next, we investigated to which extent the phase of the menstrual cycle and dienogest treatment influence the steroid correlations (Fig. 6 and Fig. S2). Dienogest has been designed to be a highly selective P4 receptor agonist and anti-androgenic activity has also been reported [21].

In serum, the following clear differences between the phases of the menstrual cycle are observed (Fig. 6). During the menstrual phase, three main large clusters are formed. The first cluster consists mainly of corticoids such as 11-deoxycorticosterone (11-DOC), 11-deoxycortisol, corticosterone, cortisol, and 11OHA4. A second cluster contains primarily androgens such as DHT and T and their precursors such as DHEA, A4, P5, and 17α-hydroxypregnenolone (17OHP5). There is no obvious functional association of the steroids in the last cluster such as DHEA-S; pregnanetriol (Ptriol); and 5aDIONE.

During the proliferative phase, a large cluster containing essentially all the C11-oxy steroids is identified. A second large cluster contains the steroids of the main pathways, covering the conversion of the universal precursors P5 and 17OHP5 to the end-points cortisol (glucocorticoids), corticosterone (mineralocorticoids), and DHT (androgens). A small, highly correlating cluster containing only P4 and its direct metabolites 17OHP4 and 11αOHP4 stands out.

The secretory phase is dominated by a large cluster that contains P4 and most of its metabolites. An additional cluster contains a large number of C11-oxy steroids (e.g., 11-deoxycortisol, 11-DOC, 11KA4, 11KT), whereas most of the androgens such as DHEA-S, DHEA, DHT, T, A4, and An form the last large cluster.

The correlation heat map obtained for the dienogest group is dominated by three clusters: C11-oxy androgens (11KA4; 11KT; 11OH5αDIONE; 11KDHT;11OHT), P4 and its metabolites, and a small number of corticoids (11-deoxycortisol, cortisol, corticosterone, cortisone).

Interestingly, the correlation heat maps in PF (Fig. S2, Supporting Information) show different features than in serum; in general, correlation clusters and correlation coefficients are smaller.

The menstrual phase is dominated by two clusters: one containing primarily P4 metabolites and a second one containing primarily corticoids and some C11-oxy androgens. The proliferative phase also shows strong clustering of C11-oxy androgens such as 11KT, 11KA4, 11KDHT, and 11OHT which is clearly separated from another cluster that contains the “classical” androgens A4, DHT, and T; this is similar to serum even though less pronounced. A third cluster features the corticoids such as cortisol, cortisone, corticosterone, 11-deoxycortisol, and 11-deoxycorticosterone.

The secretory phase shows two large clusters: one with P4 and its metabolites and one with corticoids and androgens. Dienogest treatment results in a small cluster with classical androgens (DHT, T, A4); a cluster with corticoids such as 11-deoxycortisol, 11-deoxycorticosterone, cortisol, corticosterone, and cortisone; and a mixed cluster that contains primarily androgens (e.g., 11OHA4, 11KT, DHEA, DHEA-S) and progestogens (e.g., P4, DHP4, 20aOHP4).

## Discussion

In the present work, we developed and validated a method for quantification of steroid hormones in human serum and PF by LC-HRMS. The method satisfyingly met the FDA criteria in terms of accuracy, precision, robustness, and stability, covering the range of clinically expected steroid concentrations for 17 analytes. Whereas targeted analysis of steroids by LC–MS/MS is already commonly employed in clinical laboratories, our setup also enables untargeted analysis of steroids and other compounds not covered by the targeted analysis. The untargeted analysis workflow is based on an in-house generated steroid database based on authentic standards covering retention times, exact mass, and high-resolution MS spectra of 34 steroids. Absolute quantification is also ensured for these additional compounds by the use of surrogate calibrations in combination with compound-specific response factors. To the best of our knowledge, this is also the first time that comprehensive steroid profiling was applied to PF. Based on the performance metrics of the method, the surrogate calibration approach could also be employed for robust relative quantification in case an authentic standard is not available.

The differences between PF and serum are governed by the origin of the respective steroid class: while ovary-derived progestagens are higher in PF, adrenal steroids are higher in serum. An even stronger shift towards progestagens in PF during the secretory phase (while all other classes remain essentially unchanged) substantiates this assumption. The PF-serum equilibrium is most probably influenced by the peritoneum, as this semi-permeable membrane can impose an inertia and delay for the onset of the ovarian steroids and P4 peaks in the serum.

Investigation of the PF steroid microenvironment allowed us to gain additional insight into local steroid metabolism. A decreased cortisol/cortisone ratio in PF suggests high activity of 11βHSD2 in the endometrium, for which P4 metabolites are also substrates. 11βHSD2 is responsible for the deactivation of cortisol and has been reported to be expressed in high concentrations in the placenta and endometrium; during pregnancy, it is thought to be crucial for the protection of the fetus from high circulating levels of maternal glucocorticoids [22]. Our finding of higher 11βHSD2 activity in PF compared to serum substantiates this assumption. C11-oxy androgen pathways seem to be most active during the proliferative phase as evidenced by the strongest correlations between 11KA4, 11KT, 11OHT, 11KDHT, and 11K5αDIONE, resulting in a large correlation cluster for this compound group.

These observations match previous publications and confirm the advantage of measuring “local” bioliquids to assess specific organ function by reducing interferences from the peripheral blood. In adenomyosis, an estrogen-related process, it has been hypothesized that uterine dysfunctions may result in local hyperestrogenism with normal peripheral estradiol levels but increased levels of estradiol in menstrual blood [23]. In endometrial cancer, a significant increase in the ovarian venous levels of androstenedione and testosterone was found while the androgen levels in peripheral blood were not significantly different between cases and controls [24]. PF might therefore be especially well-suited for studying the conditions of the reproductive organs without being technically demanding in comparison to highly invasive biopsies and ovarian venous blood draws.

Using our extended steroid panel, we were able to show that treatment with dienogest leads to steroid profiles that are different from all the menstrual phases while showing some resemblance to the menstrual and proliferative phases based on the activity of C11-oxygenated steroid pathways.

However, these results should be interpreted carefully as a major limitation of our study is the small sample size and the heterogeneity in the patients’ clinical presentation. Yet, our main goal was to show the technical feasibility of our approach which we will now be able to extend to large patient cohorts. Our lab provides steroid profiling for clinical routine diagnostics for approximately 2000 patients per year. The data from these patients is a true treasure as many different diseases are represented; samples have been obtained under very standardized conditions in the clinics, and a substantial amount of additional clinical information is available. Being able to perform quantitative untargeted analysis on these samples opens completely new possibilities. We can expand our way of thinking beyond the narrow targeted view to allow investigations into novel (steroid) pathways that might better explain underlying disease or for the discovery of additional biomarkers.

## Supplementary Information

Below is the link to the electronic supplementary material.

Supplementary file1 (DOCX 2728 KB)

Supplementary file2 (XLSX 99 KB)

## Data Availability

Upon request.

## References

[1] Greaves RF, Jevalikar G, Hewitt JK, Zacharin MR (2014). A guide to understanding the steroid pathway: new insights and diagnostic implications. Clin Biochem.

[2] Miller WL, Auchus RJ (2011). The molecular biology, biochemistry, and physiology of human steroidogenesis and its disorders. Endocr Rev.

[3] Takagi K, Miki Y, Ishida T, Sasano H, Suzuki T (2018). The interplay of endocrine therapy, steroid pathways and therapeutic resistance: importance of androgen in breast carcinoma. Molecular And Cellular Endocrinology.

[4] Wudy SA, Schuler G, Sanchez-Guijo A, Hartmann MF (2017) The art of measuring steroids: principles and practice of current hormonal steroid analysis. J Steroid Biochem Mol Biol. 10.1016/J.Jsbmb.2017.09.003 10.1016/j.jsbmb.2017.09.003 28962971

[5] Eisenhofer G, Peitzsch M, Kaden D, Langton K, Pamporaki C, Masjkur J, Tsatsaronis G, Mangelis A, Williams TA, Reincke M, Lenders JWM, Bornstein SR (2017) Reference intervals for plasma concentrations of adrenal steroids measured by LC-MS/MS: impact of gender, age, oral contraceptives, body mass index and blood pressure status. Clinica Chimica Acta; International Journal Of Clinical Chemistry 470:115–124. 10.1016/J.Cca.2017.05.002 10.1016/j.cca.2017.05.002 PMC5504266 28479316

[6] Keevil BG (2016). LC-MS/MS analysis of steroids in the clinical laboratory. Clin Biochem.

[7] Koal T, Schmiederer D, Pham-Tuan H, Rohring C, Rauh M (2012). Standardized LC-MS/MS based steroid hormone profile-analysis. J Steroid Biochem Mol Biol.

[8] Hines JM, Bancos I, Bancos C, Singh RD, Avula AV, Young WF, Grebe SK, Singh RJ (2017) High-resolution, accurate-mass (HRAM) mass spectrometry urine steroid profiling in the diagnosis of adrenal disorders. Clin Chem. 10.1373/Clinchem.2017.271106 10.1373/clinchem.2017.271106 28814383

[9] Travers S, Martinerie L, Bouvattier C, Boileau P, Lombes M, Pussard E (2017). Multiplexed steroid profiling of gluco- and mineralocorticoids pathways using a liquid chromatography tandem mass spectrometry method. J Steroid Biochem Mol Biol.

[10] Hunter RH, Cicinelli E, Einer-Jensen N (2007). Peritoneal fluid as an unrecognised vector between female reproductive tissues. Acta Obstet Gynecol Scand.

[11] Donnez J, Langerock S, Thomas K (1982). Peritoneal fluid volume and 17 beta-estradiol and progesterone concentrations in ovulatory, anovulatory, and postmenopausal women. Obstetrics And Gynecology.

[12] Kim-Björklund T, Landgren BM, Hamberger L (1991). Peritoneal fluid volume and levels of steroid hormones and gonadotrophins in peritoneal fluid of normal and norethisterone-treated women. Human Reproduction (Oxford, England).

[13] Xu X, Othman Eel D, Issaq HJ, Hornung D, Al-Hendy A, Veenstra TD (2008). Multiplexed quantitation of endogenous estrogens and estrogen metabolites in human peritoneal fluid. Electrophoresis.

[14] Huhtinen K, Saloniemi-Heinonen T, Keski-Rahkonen P, Desai R, Laajala D, Ståhle M, Häkkinen MR, Awosanya M, Suvitie P, Kujari H, Aittokallio T, Handelsman DJ, Auriola S, Perheentupa A, Poutanen M (2014). Intra-tissue steroid profiling indicates differential progesterone and testosterone metabolism in the endometrium and endometriosis lesions. The Journal Of Clinical Endocrinology And Metabolism.

[15] Forsse D, Tangen IL, Fasmer KE, Halle MK, Viste K, Almås B, Bertelsen BE, Trovik J, Haldorsen IS, Krakstad C (2020). Blood steroid levels predict survival in endometrial cancer and reflect tumor estrogen signaling. Gynecol Oncol.

[16] Blanco LZ, Jr., Kuhn E, Morrison JC, Bahadirli-Talbott A, Smith-Sehdev A, Kurman RJ (2017) Steroid hormone synthesis by the ovarian stroma surrounding epithelial ovarian tumors: a potential mechanism in ovarian tumorigenesis. Modern Pathology : An Official Journal Of The United States And Canadian Academy Of Pathology, Inc 30 (4):563–576. 10.1038/Modpathol.2016.219 10.1038/modpathol.2016.219 28059101

[17] Ose J, Poole EM, Schock H, Lehtinen M, Arslan AA, Zeleniuch-Jacquotte A, Visvanathan K, Helzlsouer K, Buring JE, Lee IM, Tjønneland A, Dossus L, Trichopoulou A, Masala G, Onland-Moret NC, Weiderpass E, Duell EJ, Idahl A, Travis RC, Rinaldi S, Merritt MA, Trabert B, Wentzensen N, Tworoger SS, Kaaks R, Fortner RT (2017). Androgens are differentially associated with ovarian cancer subtypes in the Ovarian Cancer Cohort Consortium. Can Res.

[18] U.S. Department of Health and Human Services Food and Drug Administration Center for Drug Evaluation and Research (CDER) and Center for Veterinary Medicine (CVM) (2018) Bioanalytical Method Validation Guidance For Industry.

[19] Pang Z, Chong J, Zhou G, De Lima Morais DA, Chang L, Barrette M, Gauthier C, Jacques P-É, Li S, Xia J (2021) Metaboanalyst 5.0: narrowing the gap between raw spectra and functional insights. Nucleic Acids Research 49 (W1):W388-W396. 10.1093/Nar/Gkab382 10.1093/nar/gkab382 PMC8265181 34019663

[20] Wang Q, Mesaros C, Blair IA (2016). Ultra-high sensitivity analysis of estrogens for special populations in serum and plasma by liquid chromatography–mass spectrometry: assay considerations and suggested practices. The Journal Of Steroid Biochemistry And Molecular Biology.

[21] Ruan X, Seeger H, Mueck AO (2012). The pharmacology of dienogest. Maturitas.

[22] Smith RE, Salamonsen LA, Komesaroff PA, Li KX, Myles KM, Lawrence M, Krozowski Z (1997). 11 Beta-hydroxysteroid dehydrogenase type II in the human endometrium: localization and activity during the menstrual cycle. The Journal Of Clinical Endocrinology And Metabolism.

[23] Vannuccini S, Tosti C, Carmona F, Huang SJ, Chapron C, Guo SW, Petraglia F (2017). Pathogenesis of adenomyosis: an update on molecular mechanisms. Reprod Biomed Online.

[24] Akhmedkhanov A, Zeleniuch-Jacquotte A, Toniolo P (2001). Role of exogenous and endogenous hormones in endometrial cancer: review of the evidence and research perspectives. Ann N Y Acad Sci.

