# Supplementary material for: Parallel targeted and non-targeted quantitative analysis of steroids in human serum and peritoneal fluid by liquid chromatography high-resolution mass spectrometry
Source: Anal Bioanal Chem. 2022 Jan 19;414(25):7461–72. doi: 10.1007/s00216-022-03881-3 (PMC9482906; doi:10.1007/s00216-022-03881-3)
Supplement: Supplementary file 1 — Supplementary file1 (DOCX 2728 KB) [file 216_2022_3881_MOESM1_ESM.docx]

**Parallel targeted and non-targeted quantitative analysis of steroids in human serum and peritoneal fluid by liquid chromatography high-resolution mass spectrometry**

Thomas Andrieu^1,2^, Therina du Toit^1^, Bruno Vogt^3^, Michael D. Mueller^1,2^, Michael Groessl^3^

^1^ Department of Biomedical Research (DBMR), University of Bern, Bern, Switzerland.

^2^ Department of Gynecology and Gynecological Oncology, Inselspital, Bern University Hospital, University of Bern, Bern, Switzerland.

^3^ Department of Nephrology and Hypertension, Inselspital, Bern University Hospital, University of Bern, Bern, Switzerland.

**Corresponding author:**

Michael Groessl, PhD

Department of Nephrology and Hypertension

Inselspital, Bern University Hospital, and University of Bern

Freiburgstrasse, 3010 Bern, Switzerland

tel: +41 31 632 29477, e-mail: michael.groessl@dbmr.unibe.ch

**Supporting Information**

**Table S1.** Targeted analytes, including retention time (RT), measurement mode and m/z value used for quantification.

| ***Analytes*** | | | | |  | ***Internal Standards*** | | | |
| --- | --- | --- | --- | --- | --- | --- | --- | --- | --- |
| **Compound Name** | **Abbr.** | **RT (min)** | **Mode** | **Signal (m/z)** |  | **Compound Name** | **RT (min)** | **Mode** | **Signal (m/z)** |
| Aldosterone | Aldo | 5.47 | Full Scan | 359.1864 |  | Aldosterone D4 | 5.44 | Full Scan | 363.2115 |
| Cortisone | E | 6.10 | PRM | 361.2010 > 163.1112 |  | Cortisone D8 | 6.04 | Full Scan | 369.2512 |
| Cortisol | F | 6.64 | Full Scan | 363.2166 |  | Cortisol D4 | 6.61 | Full Scan | 367.2417 |
| DHEA-S | DHEA-S | 7.71 | Full Scan | 271.2056 |  | DHEA-S D5 | 7.66 | Full Scan | 276.2370 |
| 21-Deoxycortisol | 21dF | 7.91 | PRM | 347.2217 > 121.0647 |  | 21-Deoxycortisol D8 | 7.86 | Full Scan | 355.2719 |
| Corticosterone | CORT | 8.38 | Full Scan | 347.2217 |  | Corticosterone D4 | 8.35 | Full Scan | 351.2468 |
| 11-Deoxycortisol | S | 8.76 | PRM | 347.2217 > 109.0648 |  | 11-Deoxycortisol D5 | 8.71 | Full Scan | 352.2531 |
| Androstenedione | A4 | 9.88 | PRM | 287.2006 > 109.0648 |  | Androstenedione 13C3 | 9.86 | Full Scan | 290.2106 |
| 11-Deoxycorticosterone | DOC | 10.46 | PRM | 331.2268 > 109.0648 |  | 11-Deoxycorticosterone D8 | 10.36 | Full Scan | 339.2770 |
| Testosterone | T | 10.54 | PRM | 289.2162 > 109.0648 |  | Testosterone D3 | 10.51 | Full Scan | 292.2350 |
| Dehydroepiandrosterone | DHEA | 10.99 | Full Scan | 271.2056 |  | 17α-Hydroxyprogesterone D8 | 11.12 | Full Scan | 339.2770 |
| 17α-Hydroxyprogesterone | 17OHP4 | 11.20 | Full Scan | 331.2268 |  | Epiandrosterone D4 | 11.71 | Full Scan | 259.2360 |
| Dihydrotestosterone | DHT | 12.17 | Full Scan | 291.2319 |  | Dihydrotestosterone D3 | 12.07 | Full Scan | 294.2507 |
| Etiocholanolone | Etio | 13.22 | Full Scan | 255.2101 |  | Progesterone D9 | 13.17 | Full Scan | 324.2884 |
| Progesterone | P4 | 13.27 | PRM | 315.2319 > 109.0648 |  | Pregnenolone D4 | 13.97 | Full Scan | 303.2621 |
| Androsterone | Andro | 13.41 | Full Scan | 255.2094 |  |  |  |  |  |
| Pregnenolone | P5 | 14.06 | Full Scan | 299.2369 |  |  |  |  |  |

**Table S2.** Concentration of calibrants 1-12 and QC samples; all values in nmol/L (n=18).

|  | **QC high** | **QC mid** | **QC low** | **QC LLOQ** | **1** | **2** | **3** | **4** | **5** | **6** | **7** | **8** | **9** | **10** | **11** | **12** |
| --- | --- | --- | --- | --- | --- | --- | --- | --- | --- | --- | --- | --- | --- | --- | --- | --- |
| **11-Deoxycorticosterone** | 189.1 | 11.82 | 0.739 | 0.092 | 189.1 | 94.6 | 47.3 | 23.6 | 11.8 | 5.91 | 2.96 | 1.478 | 0.739 | 0.369 | 0.185 | 0.092 |
| **11-Deoxycortisol** | 90.2 | 5.64 | 0.352 | 0.088 | 180.4 | 90.2 | 45.1 | 22.6 | 11.3 | 5.64 | 2.82 | 1.409 | 0.705 | 0.352 | 0.176 | 0.088 |
| **17α-Hydroxyprogesterone** | 94.6 | 5.91 | 0.369 | 0.092 | 189.1 | 94.6 | 47.3 | 23.6 | 11.8 | 5.91 | 2.96 | 1.478 | 0.739 | 0.369 | 0.185 | 0.092 |
| **21-Deoxycortisol** | 180.4 | 11.27 | 0.705 | 0.088 | 180.4 | 90.2 | 45.1 | 22.6 | 11.3 | 5.64 | 2.82 | 1.409 | 0.705 | 0.352 | 0.176 | 0.088 |
| **5α-Dihydrotestosterone** | 107.6 | 6.72 | 0.420 | 0.105 | 215.2 | 107.6 | 53.8 | 26.9 | 13.4 | 6.73 | 3.36 | 1.681 | 0.841 | 0.42 | 0.21 | 0.105 |
| **Aldosterone** | 173.4 | 10.84 | 0.677 | 0.085 | 173.4 | 86.7 | 43.4 | 21.7 | 10.8 | 5.42 | 2.71 | 1.355 | 0.677 | 0.339 | 0.169 | 0.085 |
| **Androstenedione** | 109.1 | 6.82 | 0.426 | 0.107 | 218.2 | 109.1 | 54.6 | 27.3 | 13.6 | 6.82 | 3.41 | 1.705 | 0.852 | 0.426 | 0.213 | 0.107 |
| **Androsterone** | 430.4 | 26.90 | 1.681 | 0.42 | 860.8 | 430.4 | 215.2 | 107.6 | 53.8 | 26.90 | 13.45 | 6.725 | 3.362 | 1.681 | 0.841 | 0.42 |
| **Corticosterone** | 721.6 | 45.10 | 2.819 | 0.705 | 1443.2 | 721.6 | 360.8 | 180.4 | 90.2 | 45.10 | 22.55 | 11.275 | 5.637 | 2.819 | 1.409 | 0.705 |
| **Cortisol** | 8966.5 | 560.41 | 35.025 | 0.378 | 8966.5 | 4483.3 | 2241.6 | 1120.8 | 560.4 | 280.20 | 140.10 | 35.026 | 8.756 | 2.189 | 0.547 | 0.137 |
| **Cortisone** | 1387.2 | 86.70 | 5.419 | 0.177 | 1387.2 | 693.6 | 346.8 | 173.4 | 86.7 | 43.35 | 21.68 | 7.225 | 2.408 | 0.803 | 0.268 | 0.089 |
| **DHEA** | 433.4 | 27.09 | 1.693 | 0.846 | 866.8 | 433.4 | 216.7 | 108.3 | 54.2 | 27.09 | 13.54 | 6.772 | 3.386 | 1.693 | 0.846 | 0.423 |
| **DHEA-S** | 12805.1 | 800.32 | 50.020 | 6.252 | 12805.1 | 6402.5 | 3201.3 | 1600.6 | 800.3 | 400.16 | 200.08 | 100.04 | 50.02 | 25.01 | 12.505 | 6.252 |
| **Etiocholanolone** | 215.2 | 13.45 | 0.841 | 0.21 | 215.2 | 107.6 | 53.8 | 26.9 | 13.4 | 6.73 | 3.36 | 1.681 | 0.841 | 0.42 | 0.21 | 0.105 |
| **Pregnenolone** | 789.9 | 49.37 | 3.086 | 0.771 | 789.9 | 395.0 | 197.5 | 98.7 | 49.4 | 24.69 | 12.34 | 6.171 | 3.086 | 1.543 | 0.771 | 0.386 |
| **Progesterone** | 795.0 | 49.69 | 3.106 | 0.476 | 1590.0 | 795.0 | 397.5 | 198.8 | 99.4 | 49.69 | 24.84 | 12.422 | 6.211 | 3.106 | 1.553 | 0.476 |
| **Testosterone** | 433.4 | 27.09 | 1.693 | 0.105 | 866.8 | 433.4 | 216.7 | 108.3 | 54.2 | 27.09 | 13.54 | 6.772 | 3.386 | 1.693 | 0.846 | 0.105 |

**Table S3.** Intra-day accuracy and precision (n=6 at each level). Recovery obtained by comparing the results of extracted samples with corresponding extracts of blanks spiked with the analyte post-extraction.

|  | **Accuracy (RSD, %)** | | |  | **Precision (relative error, %)** | | | **Recovery (%)** |
| --- | --- | --- | --- | --- | --- | --- | --- | --- |
|  | *High* | *Mid* | *Low* |  | *High* | *Mid* | *Low* | *Mid* |
| **11-Deoxycorticosterone** | 3.2 | 5.6 | 5.0 |  | -1.2 | -1.7 | -3.2 | 93 |
| **11-Deoxycortisol** | 5.5 | 4.4 | 6.0 |  | 2.9 | -3.6 | 0.8 | 91 |
| **17α-Hydroxyprogesterone** | 5.8 | 7.4 | 6.9 |  | -7.6 | 0.6 | 3.7 | 94 |
| **21-Deoxycortisol** | 9.3 | 7.6 | 8.3 |  | -2.9 | -4.9 | 3.1 | 87 |
| **5α-Dihydrotestosterone** | 7.0 | 6.5 | 4.9 |  | -2.9 | -4.2 | 7.3 | 89 |
| **Aldosterone** | 8.9 | 5.8 | 0.0 |  | -6.7 | 3.2 | 3.3 | 99 |
| **Androstenedione** | 6.8 | 6.9 | 7.5 |  | -3.4 | 3.7 | 2.4 | 92 |
| **Androsterone** | 6.7 | 5.4 | 6.4 |  | 0.4 | 3.3 | 3.3 | 85 |
| **Corticosterone** | 5.7 | 7.4 | 7.1 |  | 0.3 | -4.4 | 0.9 | 86 |
| **Cortisol** | 6.9 | 7.8 | 6.1 |  | -7.2 | 0.9 | 3.4 | 92 |
| **Cortisone** | 5.7 | 4.0 | 4.9 |  | -1.4 | -2.1 | 3.9 | 93 |
| **DHEA** | 8.8 | 4.0 | 9.8 |  | 1.7 | -6.3 | 9.1 | 90 |
| **DHEA-S** | 7.1 | 12.9 | 12.2 |  | -0.5 | -11.1 | 5.8 | 80 |
| **Etiocholanolone** | 6.0 | 7.1 | 6.5 |  | 0.9 | 4.0 | 0.1 | 95 |
| **Pregnenolone** | 7.3 | 5.5 | 3.7 |  | 0.0 | -5.2 | 6.5 | 87 |
| **Progesterone** | 5.4 | 4.7 | 4.7 |  | -1.0 | 0.2 | 4.7 | 88 |
| **Testosterone** | 5.4 | 6.3 | 3.7 |  | 0.1 | -5.5 | 3.2 | 95 |

**Table S4.** Robustness accessed by accuracy and precision of the assay performed by a different technician and different lots of solvents, SPE plate and solvents (n=6 at each level).

|  | **Accuracy (RSD, %)** | | |  | **Precision (relative error, %)** | | |
| --- | --- | --- | --- | --- | --- | --- | --- |
|  | *High* | *Mid* | *Low* |  | *High* | *Mid* | *Low* |
| **11-Deoxycorticosterone** | 3.3 | 2.8 | 10.1 |  | -2.3 | 13.5 | 9.2 |
| **11-Deoxycortisol** | 4.9 | 3.3 | 6.6 |  | 4.6 | 0.3 | 7.8 |
| **17α-Hydroxyprogesterone** | 7.1 | 8.4 | 12.2 |  | -6.8 | 9.9 | 9.3 |
| **21-Deoxycortisol** | 1.3 | 2.3 | 6.6 |  | 4.2 | 12.7 | 13.5 |
| **5α-Dihydrotestosterone** | 3.9 | 3.4 | 9.3 |  | 7.6 | 8.7 | 7.7 |
| **Aldosterone** | 3.5 | 2.8 | 9.2 |  | 6.7 | 1.9 | 7.1 |
| **Androstenedione** | 6.0 | 2.8 | 5.7 |  | 7.6 | 6.9 | 8.5 |
| **Androsterone** | 4.2 | 3.4 | 7.6 |  | 8.5 | 4.1 | 10.5 |
| **Corticosterone** | 6.2 | 1.5 | 7.7 |  | 12.5 | 3.3 | 14.2 |
| **Cortisol** | 5.2 | 3.1 | 7.6 |  | 13.1 | 3.6 | 12.2 |
| **Cortisone** | 5.2 | 9.0 | 3.1 |  | 5.9 | 14.2 | 12.4 |
| **DHEA** | 2.3 | 3.5 | 5.9 |  | 7.0 | 4.8 | 13.1 |
| **DHEA-S** | 2.9 | 1.7 | 10.4 |  | 7.9 | 9.0 | -4.8 |
| **Etiocholanolone** | 7.5 | 7.1 | 9.4 |  | 12.1 | 4.3 | -8.9 |
| **Pregnenolone** | 5.5 | 7.0 | 9.6 |  | 8.0 | 10.0 | 7.8 |
| **Progesterone** | 3.7 | 3.1 | 9.2 |  | 6.2 | 13.2 | 8.9 |
| **Testosterone** | 4.4 | 1.9 | 8.0 |  | 9.5 | -3.1 | 8.6 |

**Table S5.** Freeze-thaw stability (n=6 at each level).

|  | **Accuracy (RSD, %)** | | |  | **Precision (relative error, %)** | | |
| --- | --- | --- | --- | --- | --- | --- | --- |
|  | *High* | *Mid* | *Low* |  | *High* | *Mid* | *Low* |
| **11-Deoxycorticosterone** | 3.5 | 5.6 | 7.9 |  | 9.6 | 9.6 | 14.6 |
| **11-Deoxycortisol** | 4.4 | 4.7 | 11.1 |  | 8.4 | 11.2 | 6.5 |
| **17α-Hydroxyprogesterone** | 2.3 | 5.5 | 11.1 |  | 10.3 | 11.8 | -11.5 |
| **21-Deoxycortisol** | 2.3 | 7.2 | 11.5 |  | 6.7 | 5.7 | 13.9 |
| **5α-Dihydrotestosterone** | 1.5 | 6.4 | 7.6 |  | 13.7 | 14.6 | 11.6 |
| **Aldosterone** | 14.6 | 5.9 | 3.3 |  | 10.3 | 5.7 | 6.3 |
| **Androstenedione** | 9.1 | 3.4 | 4.4 |  | 5.0 | -2.6 | -3.4 |
| **Androsterone** | 4.4 | 13.1 | 5.5 |  | 9.2 | -2.3 | 6.8 |
| **Corticosterone** | 6.7 | 4.8 | 0.5 |  | 7.7 | 14.4 | 7.6 |
| **Cortisol** | 6.9 | 0.9 | 1.2 |  | -4.3 | 9.2 | 9.5 |
| **Cortisone** | 3.4 | 6.2 | 2.1 |  | 12.4 | 10.4 | 8.4 |
| **DHEA** | 14.4 | 9.8 | 9.9 |  | 7.6 | 7.4 | 6.5 |
| **DHEA-S** | 7.9 | 8.3 | 10.2 |  | 11.3 | 8.7 | 12.3 |
| **Etiocholanolone** | 9.6 | 8.7 | 9.7 |  | 8.8 | -6.5 | -3.3 |
| **Pregnenolone** | 12.1 | 9.8 | 10.5 |  | 13.2 | -3.7 | -9.6 |
| **Progesterone** | 3.4 | 6.9 | 4.9 |  | 8.1 | 11.5 | 2.7 |
| **Testosterone** | 5.0 | 7.5 | 4.2 |  | 7.1 | 10.3 | 10.6 |

**Table S6.** Bench-top stability (n=6 at each level).

|  | **Accuracy (RSD, %)** | | |  | **Precision (relative error, %)** | | |
| --- | --- | --- | --- | --- | --- | --- | --- |
|  | *High* | *Mid* | *Low* |  | *High* | *Mid* | *Low* |
| **11-Deoxycorticosterone** | 2.0 | 3.2 | 3.8 |  | 3.6 | -1.6 | 3.2 |
| **11-Deoxycortisol** | 6.9 | 2.9 | 6.0 |  | -5.1 | 4.8 | -6.2 |
| **17α-Hydroxyprogesterone** | 6.0 | 4.3 | 3.6 |  | 9.8 | 8.6 | -8.9 |
| **21-Deoxycortisol** | 0.5 | 6.9 | 6.2 |  | 14.3 | 5.4 | -7.3 |
| **5α-Dihydrotestosterone** | 2.6 | 3.4 | 5.7 |  | 5.4 | 4.7 | 2.3 |
| **Aldosterone** | 1.4 | 3.8 | 0.0 |  | 8.3 | 4.5 | 3.1 |
| **Androstenedione** | 11.3 | 4.3 | 1.8 |  | -3.3 | -9.2 | -5.2 |
| **Androsterone** | 2.3 | 4.3 | 2.2 |  | 4.6 | -1.8 | -8.2 |
| **Corticosterone** | 6.4 | 4.3 | 3.6 |  | -10.1 | 9.1 | 9.8 |
| **Cortisol** | 12.4 | 3.5 | 7.7 |  | 1.4 | -8.5 | -6.5 |
| **Cortisone** | 2.7 | 1.7 | 3.3 |  | 5.1 | 6.4 | 2.5 |
| **DHEA** | 7.5 | 6.3 | 9.5 |  | 2.2 | 5.3 | 1.8 |
| **DHEA-S** | 7.2 | 7.1 | 13.3 |  | -2.7 | 12.5 | 6.4 |
| **Etiocholanolone** | 2.0 | 2.6 | 6.1 |  | -3.3 | -7.0 | -2.0 |
| **Pregnenolone** | 8.7 | 3.3 | 4.6 |  | -4.2 | 9.8 | -8.2 |
| **Progesterone** | 10.9 | 1.2 | 1.9 |  | 2.3 | 2.9 | -0.3 |
| **Testosterone** | 4.6 | 1.6 | 1.4 |  | -0.3 | 0.4 | 7.4 |

**Table S7.** Autosampler stability (n=6 at each level).

|  | **Accuracy (RSD, %)** | | |  | **Precision (relative error, %)** | | |
| --- | --- | --- | --- | --- | --- | --- | --- |
|  | *High* | *Mid* | *Low* |  | *High* | *Mid* | *Low* |
| **11-Deoxycorticosterone** | 5.9 | 4.3 | 11.7 |  | -4.2 | -1.2 | -0.4 |
| **11-Deoxycortisol** | 5.4 | 5.6 | 6.1 |  | -7.3 | 0.2 | -6.3 |
| **17α-Hydroxyprogesterone** | 2.6 | 4.4 | 10.9 |  | 11.4 | 8.9 | 2.5 |
| **21-Deoxycortisol** | 9.9 | 9.8 | 8.1 |  | 1.7 | 2.4 | 6.0 |
| **5α-Dihydrotestosterone** | 9.5 | 9.7 | 7.7 |  | 3.6 | 2.2 | -9.6 |
| **Aldosterone** | 3.5 | 4.4 | 5.2 |  | -3.5 | -8.7 | -9.3 |
| **Androstenedione** | 10.6 | 7.0 | 11.0 |  | 2.4 | -5.3 | 0.8 |
| **Androsterone** | 13.8 | 11.3 | 1.7 |  | 10.8 | 4.8 | 10.0 |
| **Corticosterone** | 8.5 | 7.5 | 5.2 |  | 1.9 | 3.7 | 0.7 |
| **Cortisol** | 13.8 | 7.4 | 5.7 |  | 2.9 | 4.5 | 7.7 |
| **Cortisone** | 7.8 | 3.6 | 6.6 |  | 2.7 | 6.8 | -0.6 |
| **DHEA** | 14.1 | 11.5 | 11.7 |  | 9.2 | 6.0 | 3.8 |
| **DHEA-S** | 8.9 | 7.1 | 5.9 |  | 1.7 | 10.1 | 13.6 |
| **Etiocholanolone** | 14.4 | 6.5 | 8.5 |  | 9.5 | 8.1 | 9.7 |
| **Pregnenolone** | 8.5 | 6.5 | 5.1 |  | -7.2 | -2.2 | -8.6 |
| **Progesterone** | 6.1 | 5.0 | 8.0 |  | 6.0 | 2.8 | 0.8 |
| **Testosterone** | 10.4 | 8.2 | 6.1 |  | 12.0 | 7.3 | -13.7 |

**Table S8.** Long-term stability (n=6 at each level).

|  | **Accuracy (RSD, %)** | | |  | **Precision (relative error, %)** | | |
| --- | --- | --- | --- | --- | --- | --- | --- |
|  | *High* | *Mid* | *Low* |  | *High* | *Mid* | *Low* |
| **11-Deoxycorticosterone** | 3.7 | 4.3 | 5.3 |  | -3.8 | -1.5 | -2.7 |
| **11-Deoxycortisol** | 3.5 | 2.7 | 9.0 |  | 8.5 | 7.3 | 6.8 |
| **17α-Hydroxyprogesterone** | 5.8 | 5.9 | 4.0 |  | -1.1 | 1.5 | 6.7 |
| **21-Deoxycortisol** | 5.0 | 14.0 | 8.3 |  | 6.5 | 3.4 | -2.3 |
| **5α-Dihydrotestosterone** | 6.0 | 5.5 | 11.5 |  | 9.3 | 9.8 | 7.5 |
| **Aldosterone** | 2.3 | 5.5 | 11.1 |  | 8.4 | 11.2 | 6.5 |
| **Androstenedione** | 8.8 | 2.1 | 7.6 |  | 3.3 | 10.3 | 8.4 |
| **Androsterone** | 2.9 | 8.5 | 10.6 |  | 10.2 | 9.6 | 7.8 |
| **Corticosterone** | 6.7 | 8.5 | 6.2 |  | 4.2 | 5.4 | 1.7 |
| **Cortisol** | 7.3 | 6.9 | 9.7 |  | -4.5 | 5.2 | 14.7 |
| **Cortisone** | 4.4 | 3.3 | 3.0 |  | 6.6 | 10.4 | -1.2 |
| **DHEA** | 4.8 | 5.1 | 12.6 |  | -2.0 | 2.8 | 6.0 |
| **DHEA-S** | 6.9 | 13.2 | 11.3 |  | -5.0 | 9.8 | 8.4 |
| **Etiocholanolone** | 4.7 | 7.0 | 9.9 |  | -8.0 | 1.6 | 14.5 |
| **Pregnenolone** | 6.8 | 3.2 | 7.3 |  | -3.9 | 8.1 | -8.0 |
| **Progesterone** | 3.1 | 4.0 | 7.4 |  | 7.5 | 6.2 | -4.4 |
| **Testosterone** | 5.4 | 3.1 | 3.4 |  | 6.1 | 6.3 | 5.2 |

**Table S9.** Untargeted analytes, including retention time (RT), measurement mode, m/z value used for quantification, internal reference standard used and the calibration of the untargeted analytes in the reference matrix: reference calibrant used, concentration range (nM), goodness of fit (R^2^) and the calculated response factors.

| **Compound Name** | **Abbreviation** | **Chemical formula** | **RT (min)** | **Mode** | **Signal (m/z)** | **Internal reference standard** | **Reference calibrant** | **Calibration range** | **R^2^** | **Correction factor** |
| --- | --- | --- | --- | --- | --- | --- | --- | --- | --- | --- |
| 11-Ketoandrostenedione | 11KA4 | C19H24O3 | 6.43 | Full Scan | 301.1798 | Cortisol D4 | Androstenedione | 0.1 - 1000 | 0.9785 | 0.33 |
| 11-Ketotestosterone | 11KT | C19H26O3 | 6.96 | Full Scan | 303.1955 | Cortisol D4 | Androstenedione | 0.1 – 2000 | 0.9936 | 0.37 |
| Dienogest | - | C20H25NO2 | 7.24 | Full Scan | 312.1958 | Cortisol D4 | Androstenedione | 0.1 – 1000 | 0.9944 | 0.34 |
| 5α-Androstanetrione | 11K5αDIONE | C19H26O3 | 7.34 | Full Scan | 303.1955 | Cortisol D4 | Androstenedione | 0.1 – 2000 | 0.9933 | 2.76 |
| 11β-Hydroxyandrostenedione | 11OHA4 | C19H26O3 | 7.40 | Full Scan | 303.1955 | Cortisol D4 | Androstenedione | 0.1 – 1000 | 0.9499 | 0.30 |
| 11β-hydroxytestosterone | 11OHT | C19H28O3 | 7.96 | Full Scan | 305.2111 | Cortisol D4 | Androstenedione | 0.1 – 1000 | 0.9589 | 0.27 |
| 11-Ketoandrostanolone | 11KDHT | C19H28O3 | 8.02 | Full Scan | 305.2111 | Cortisol D4 | Androstenedione | 0.1 – 1000 | 0.9957 | 1.26 |
| 6α-Hydroxyprogesterone | 6αOHP4 | C21H30O3 | 8.49 | Full Scan | 331.2268 | Androstenedione 13C3 | Androstenedione | 0.1 – 2000 | 0.9857 | 0.33 |
| 11β-hydroxy-5α-androstane-3,17-dione | 11OH5αDIONE | C19H28O3 | 8.69 | Full Scan | 305.2111 | Androstenedione 13C3 | Androstenedione | 0.1 – 1000 | 0.9982 | 4.01 |
| 16α-Hydroxyprogesterone | 16OHP4 | C21H30O3 | 8.80 | Full Scan | 331.2268 | Androstenedione 13C3 | Androstenedione | 0.1 – 1000 | 0.9912 | 0.15 |
| 11-Ketoprogesterone | 11KP4 | C21H28O3 | 9.18 | Full Scan | 329.2111 | Androstenedione 13C3 | Androstenedione | 0.1 – 2000 | 0.9916 | 0.24 |
| 11α-Hydroxyprogesterone | 11αOHP4 | C21H30O3 | 9.22 | Full Scan | 331.2268 | Androstenedione 13C3 | Androstenedione | 0.1 – 1000 | 0.9817 | 0.81 |
| 11β-hydroxyandrosterone | 11OHAn | C19H30O3 | 9.28 | Full Scan | 271.2056 | Androstenedione 13C3 | Androstenedione | 0.1 – 2000 | 0.9988 | 1.59 |
| 11-Ketoandrosterone | 11KAn | C19H28O3 | 9.35 | Full Scan | 305.2111 | Androstenedione 13C3 | Androstenedione | 0.1 – 1000 | 0.9987 | 2.14 |
| 6α-OH-Pregnanolone | 6OHTHP | C21H34O3 | 9.52 | Full Scan | 317.2475 | Androstenedione 13C3 | Androstenedione | 0.1 – 2000 | 0.9921 | 4.02 |
| 6β-Hydroxyprogesterone | 6βOHP4 | C21H30O3 | 9.88 | Full Scan | 331.2268 | Androstenedione 13C3 | Androstenedione | 0.1 – 2000 | 0.9992 | 0.74 |
| 5α-Pregnanetrione | 11KDHP4 | C21H30O3 | 10.22 | Full Scan | 331.2268 | Androstenedione 13C3 | Androstenedione | 0.1 – 1000 | 0.9992 | 10.58 |
| Pregnanetriolone | 11KPtriol | C21H34O4 | 10.25 | Full Scan | 333.2424 | Androstenedione 13C3 | Androstenedione | 0.1 – 1000 | 0.9996 | 7.32 |
| 11β-Hydroxyprogesterone | 11βOHP4 | C21H30O3 | 10.40 | Full Scan | 333.2424 | Progesterone D9 | Progesterone | 0.1 – 2000 | 0.972 | 9.65 |
| Androstenediol | A5 | C19H30O2 | 10.56 | Full Scan | 273.2213 | Androstenedione 13C3 | Androstenedione | 0.1 - 2000 | 0.9911 | 22.67 |
| 17α,20α-Dihydroxyprogesterone | 17,20diOHP4 | C21H32O3 | 10.95 | Full Scan | 333.2424 | Progesterone D9 | Progesterone | 0.1 – 1000 | 0.9713 | 0.37 |
| 17α-Hydroxypregnenolone | 17OHP5 | C21H32O3 | 11.42 | Full Scan | 297.2213 | Progesterone D9 | Progesterone | 0.1 – 2000 | 0.9914 | 45.11 |
| 5α-androstane-3,17-dione | 5αDIONE | C19H28O2 | 11.52 | Full Scan | 289.2162 | Progesterone D9 | Progesterone | 0.1 – 2000 | 0.9936 | 11.51 |
| 17α-Hydroxypregnanolone | 17OHTHP | C21H34O3 | 12.21 | Full Scan | 299.2369 | Progesterone D9 | Progesterone | 0.1 – 1000 | 0.9838 | 19.48 |
| Androstanediol | 3αDIOL | C19H32O2 | 12.94 | Full Scan | 257.2264 | Progesterone D9 | Progesterone | 0.1 – 2000 | 0.9965 | 284.33 |
| 20α-Hydroxyprogesterone | 20αOHP4 | C21H32O2 | 13.10 | Full Scan | 317.2475 | Progesterone D9 | Progesterone | 0.1 – 1000 | 0.983 | 0.26 |
| 5α/β-pregnan-3β,20α-diol | 20OH-3βTHP | C21H36O2 | 13.80 | Full Scan | 285.2577 | Progesterone D9 | Progesterone | 0.1 – 2000 | 0.9883 | 169.65 |
| 20β-Hydroxyprogesterone | 20βOHP4 | C21H32O2 | 14.27 | Full Scan | 317.2475 | Progesterone D9 | Progesterone | 0.1 – 2000 | 0.9941 | 0.58 |
| Pregnanetriol | Ptriol | C21H36O3 | 14.41 | Full Scan | 301.2526 | Progesterone D9 | Progesterone | 0.1 – 1000 | 0.9875 | 31.01 |
| 5α/β-pregnan-3β-ol-20-one | 3βTHP | C21H3402 | 14.44 | Full Scan | 319.2632 | Progesterone D9 | Progesterone | 0.1 – 2000 | 0.989 | 46.75 |
| 5α/β-Dihydroprogesterone | 5α/βDHP4 | C21H32O2 | 14.50 | Full Scan | 317.2475 | Progesterone D9 | Progesterone | 0.1 – 2000 | 0.98915 | 9.54 |
| Pregnanolone | THP | C21H34O2 | 14.66 | Full Scan | 301.2526 | Progesterone D9 | Progesterone | 0.1 – 2000 | 0.9972 | 40.85 |
| 5α-Pregnanolone | 5αTHP | C21H34O2 | 14.77 | Full Scan | 319.2632 | Progesterone D9 | Progesterone | 0.1 – 1000 | 0.9648 | 27.06 |
| 5α/β-pregnan-3α, 20α-diol | 20OHTHP | C21H36O2 | 14.82 | Full Scan | 285.2577 | Progesterone D9 | Progesterone | 0.1 - 2000 | 0.95815 | 172.79 |

**Figure S1**. Correlation analysis comparing the quantification using targeted analysis or untargeted analysis with surrogate calibration.

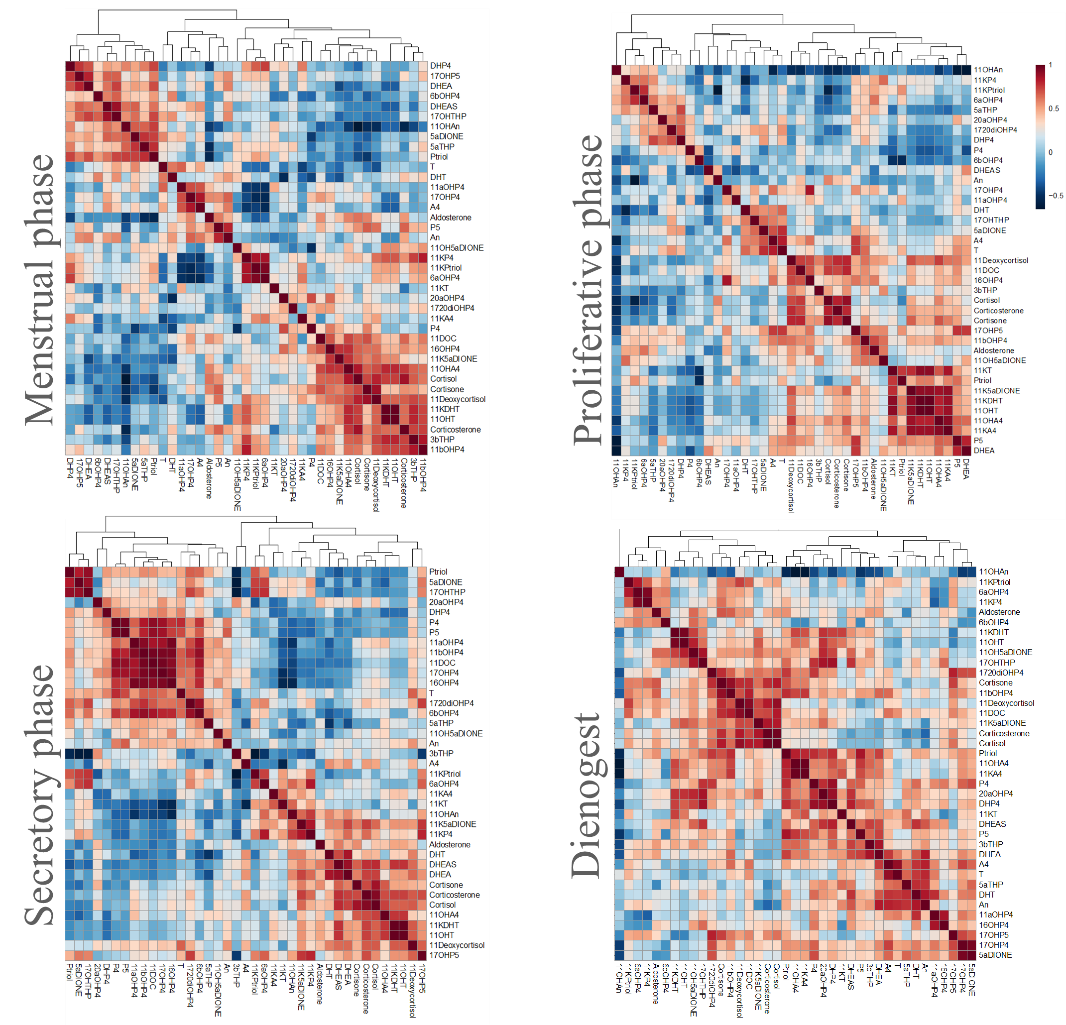


**Figure S2.** Correlation heat maps showing the correlation of all the quantified steroids (Spearman correlation) in PF during the menstrual phase, proliferative phase, secretory phase and under dienogest treatment.


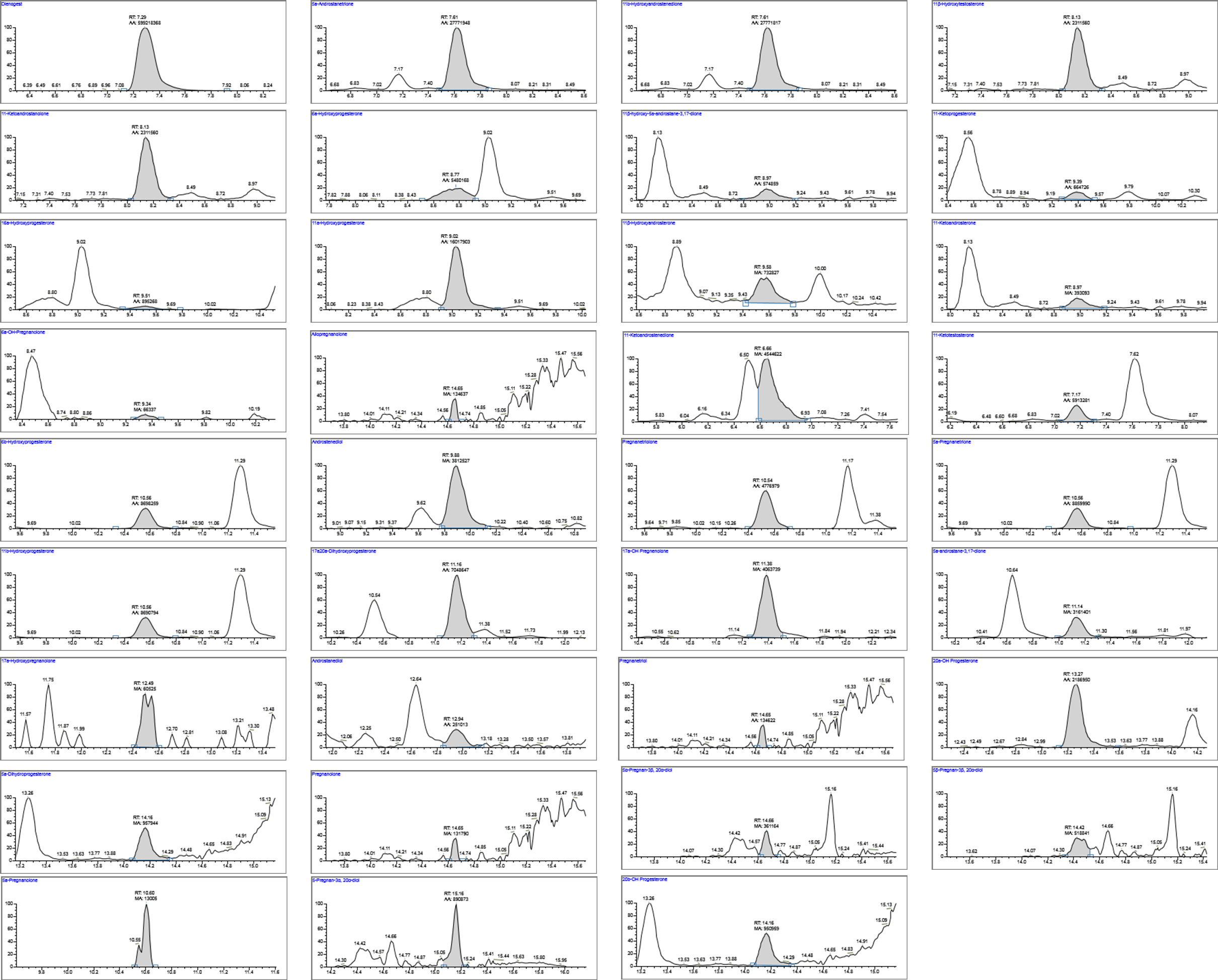


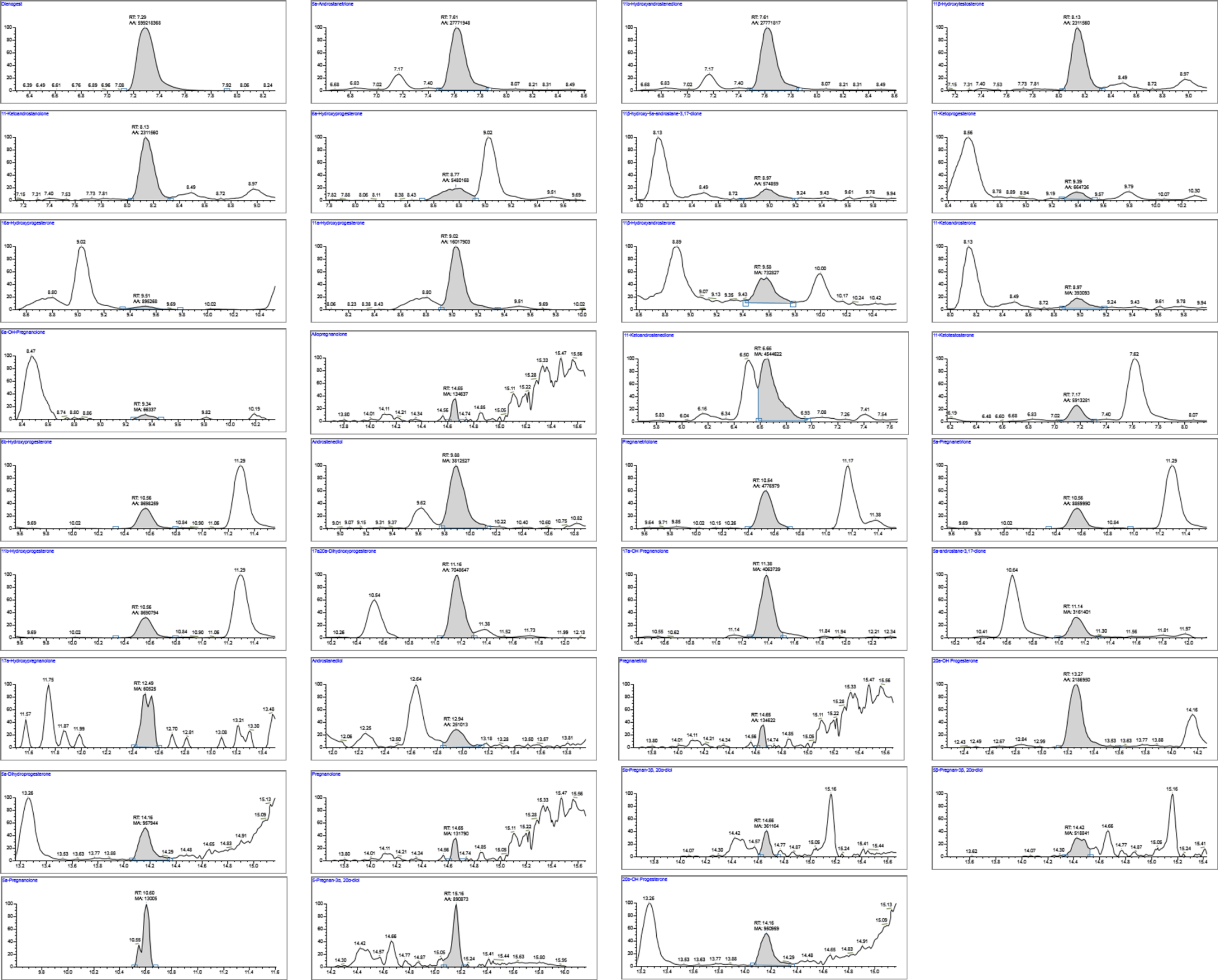


**Figure S3.** Extracted ion chromatograms from for all untargeted steroids ion a representative serum sample.
